# Circulating Extracellular Vesicles: Their Role in Patients with Abdominal Aortic Aneurysm (AAA) Undergoing EndoVascular Aortic Repair (EVAR)

**DOI:** 10.3390/ijms232416015

**Published:** 2022-12-16

**Authors:** Francesco Lorenzo Serafini, Andrea Delli Pizzi, Pasquale Simeone, Alberto Giammarino, Cristian Mannetta, Michela Villani, Jacopo Izzi, Davide Buca, Giulia Catitti, Piero Chiacchiaretta, Stefano Trebeschi, Sebastiano Miscia, Massimo Caulo, Paola Lanuti

**Affiliations:** 1 Unit of Radiology, “SS. Annunziata” Hospital, 66100 Chieti, Italy; 2 Department of Neuroscience, Imaging and Clinical Sciences, University “G. d’Annunzio”, 66100 Chieti, Italy; 3 Department of Innovative Technologies in Medicine & Dentistry, University “G. d’Annunzio”, 66100 Chieti, Italy; 4 Institute of Advanced Biomedical Technologies (ITAB), University “G. d’Annunzio”, 66100 Chieti, Italy; 5 Department of Medicine and Aging Sciences, University “G. d’Annunzio”, 66100 Chieti, Italy; 6 Center for Advanced Studies and Technologies (CAST), University “G. d’Annunzio”, 66100 Chieti, Italy; 7 Unit of Vascular Surgery, “SS. Annunziata” Hospital, 66100 Chieti, Italy; 8 Department of Radiology, Netherlands Cancer Institute, 1066 CX Amsterdam, The Netherlands

**Keywords:** extracellular vesicles, abdominal aortic aneurysm, EVAR, radiology, radiovesicolomics, AAA, endovascular aortic repair, interventional radiology

## Abstract

Abdominal aortic aneurysm (AAA) is a frequent aortic disease. If the diameter of the aorta is larger than 5 cm, an open surgical repair (OSR) or an endovascular aortic repair (EVAR) are recommended. To prevent possible complications (i.e., endoleaks), EVAR-treated patients need to be monitored for 5 years following the intervention, using computed tomography angiography (CTA). However, this radiological method involves high radiation exposure in terms of CTA/year. In such a context, the study of peripheral-blood-circulating extracellular vesicles (pbcEVs) has great potential to identify biomarkers for EVAR complications. We analyzed several phenotypes of pbcEVs using polychromatic flow cytometry in 22 patients with AAA eligible for EVAR. From each enrolled patient, peripheral blood samples were collected at AAA diagnosis, and after 1, 6, and 12 months following EVAR implantation, i.e. during the diagnostic follow-up protocol. Patients developing an endoleak displayed a significant decrease in activated-platelet-derived EVs between the baseline condition and 6 months after EVAR intervention. Furthermore, we also observed, that 1 month after EVAR implantation, patients developing an endoleak showed higher concentrations of activated-endothelial-derived EVs than patients who did not develop one, suggesting their great potential as a noninvasive and specific biomarker for early identification of EVAR complications.

## 1. Introduction

Abdominal aortic aneurysm (AAA), one of the most frequent causes of aortic diseases, is defined as a dilation or widening of the aorta to greater than 3.0 cm, which is usually more than two standard deviations above the mean aortic diameter of healthy adult men [1,2,3]. These aneurysms are most commonly fusiform in shape rather than saccular [4]. When the AAA diameter is larger than 5 cm, an elective intervention based on open surgical repair (OSR) or on endovascular aortic repair (EVAR) is recommended [5]. The imaging techniques employed to study AAA are ultrasonography (US), contrast enhanced ultrasonography (CEUS), and computed tomography angiography (CTA). In addition to their key role in pre-operative planning, they are also used during the early and late post-intervention follow-up, when it is mandatory to verify both the technical success of the therapeutic treatment, and the onset of possible complications [4] such as endoleak development. An endoleak is a persistent blood perfusion within the excluded aortic aneurysmatic sac undergoing EVAR. Endoleaks develop immediately or after the EVAR intervention have been classified into five categories by the Society for Vascular Surgery, according to their pathophysiological mechanisms (Table 1). Type II endoleak is the most diagnosed endoleak subtype. Usually, it does not require any endovascular or surgical intervention, given that it is associated with a satisfying rate of spontaneous resolution, ranging from 35.4% to 80% [6,7]. Type I and III are considered high-flow endoleaks and thus require prompt treatment due to the high risk of sac rupture. It must be underlined, that even if the employment of CEUS and CTA for the identification of possible AAA post-treatment-complication onset is considered the gold standard, these methods expose patients to high radiation in terms of CTA/year [4]. Therefore, the identification of safer biological markers for EVAR complications is needed to change the diagnostic and prognostic algorithms for these patients. In such a context, the study of peripheral blood circulating extracellular vesicles (EVs) has great potential. Extracellular vesicles (EVs) are cell derived particles carrying biological messages across biological barriers, and thereby acting as mediators of intercellular communication [8,9]. Traditionally, EVs have been classified into three subtypes, exosomes, microvesicles (or microparticles), and apoptotic bodies, based on their size or biogenesis. More recently, the International Society of Extracellular Vesicles (ISEV) recommended use of the term “extracellular vesicles” as an umbrella term for the identification of all EV subtypes [9,10,11,12,13,14,15]. EVs are constitutively produced and released into the extracellular milieu by all cell types under the effect of different stimuli, and are involved in many pathophysiological events, such as inflammation, hypoxia, oxidative or shear stress, or senescence [16,17,18,19,20,21]. Their cargoes, including lipid mediators, proteins, and genetic material, correspond to the characteristics and the status of the parental cell [22,23,24]. EVs have been implicated in many clinical conditions, such as carcinogenesis, tumor invasion/metastasis and cardiovascular dysfunction [25,26,27,28,29,30,31,32,33,34,35,36,37,38,39,40,41,42,43,44,45,46]. Furthermore, over the last few decades, EVs have been proposed as reliable biomarkers for the diagnosis and monitoring of a large number of human diseases, such as coronary artery disease [47]; neurodegenerative [48], renal [49], liver [50], and autoimmune [51] diseases; systemic sclerosis [52]; urological [53], hepatobiliary [54], and hematological [55] malignancies; and breast [56], lung [57], and ovarian cancers [58]; as well as for the monitoring of cancer outcome and treatments [59,60,61,62]. Studies on the potential of EVs as biomarkers in vascular pathology and in AAA patients are needed. Against that background, here we aimed to evaluate the potential of different phenotypes of peripheral blood EVs (i.e., endothelial and platelet derived EVs) as circulating diagnostic/prognostic biomarkers in patients affected by AAA and eligible for EVAR intervention. To this end, CEUS and CTA radiological findings were correlated with peripheral blood analyses of several EV phenotypes analyzed using polychromatic flow cytometry.

## 2. Results

### 2.1. A Stable Aneurysm Diameter Is Associated with Persistence of Endoleaks

**Figure 1:**
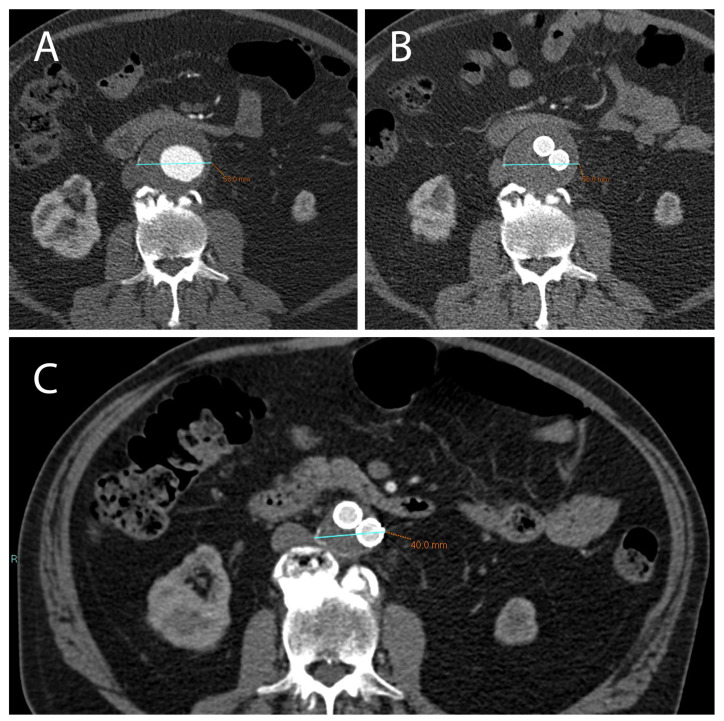
(**A**) CTA axial images show an AAA with a diameter >5.5 cm (in this case 5.8 cm) which can be treated with EVAR. (**B**) After 1 month following EVAR implantation, CTA was used to assess endoleak occurrence; of note, there was no significant reduction in AAA diameter in this short time-range. (**C**) After 12 months following EVAR intervention, a correct aneurysmatic sac exclusion without endoleak led to a significant reduction in AAA diameter (in this case the aneurysm diameter went from 5.8 cm to 4 cm).

**Figure 2:**
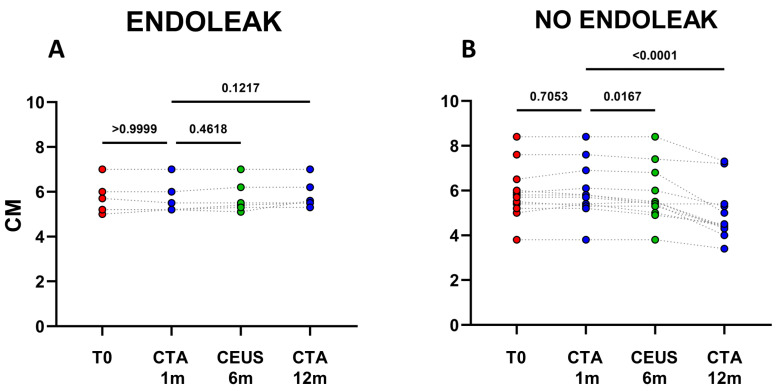
Dots represent size (cm) of aneurysms in patients developing endoleaks (*n* = 6) (**A**), and in patients who did not develop endoleaks (*n* = 12) (**B**). Patients were analyzed at baseline (T0) and after 1, 6, and 12 months following EVAR intervention, as established in the clinical protocol.

**Figure 3:**
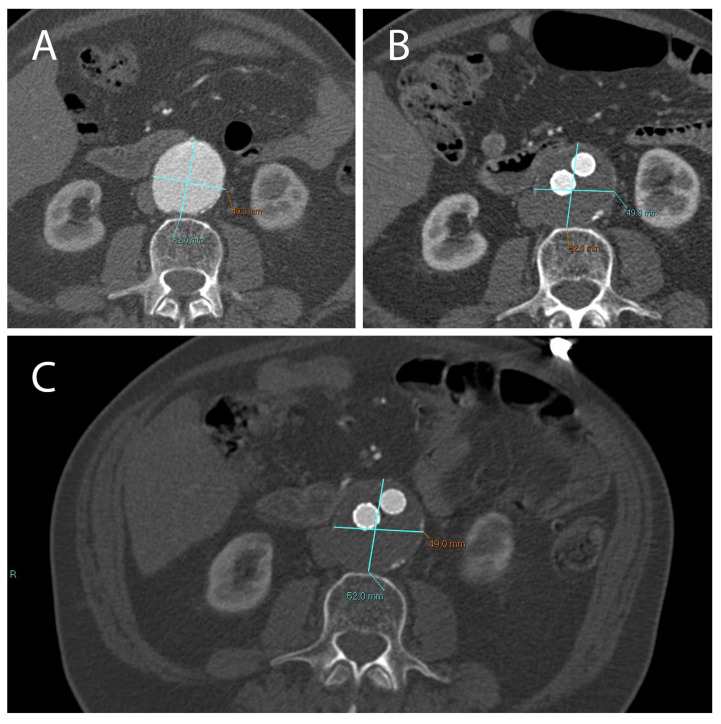
A not correctly excluded AAA treated with EVAR, with the presence of an endoleak. Treatment did not result in a reduction in aneurysm diameter compared to the pre-EVAR CTA (**A**). CT images obtained 1 month (**B**) and 12 months (**C**) after EVAR implantation.

The analysis of clinical data confirmed, that in the cohort of patients not displaying endoleaks a significant decrease in AAA size occurred between 1 and 12 months after EVAR implantation (mean decrease ~0.98 cm; *p* < 0.0001) (Figure 1 and Figure 2B). In patients with endoleak presence and persistence on the other hand, no statistical AAA size variations were registered during the same time range (Figure 2A and Figure 3).

### 2.2. Extracellular Vesicle Release in Patients Undergoing EVAR Implantation

**Figure 4:**
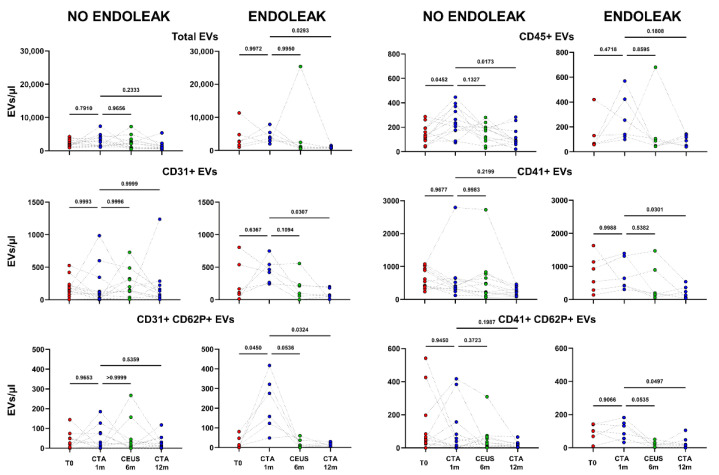
Dots represent numbers (EVs/μL) of EVs from peripheral blood of patients sorted into the category “No Endoleak” (*n* = 12) and of “Endoleak” patients (*n* = 6), analyzed at baseline (T0), and after 1, 6, and 12 months following EVAR intervention, as established in the diagnostic protocol. Each line connects the time points of a single donor. Repeated measures one-way ANOVA or a mixed-effects model was used to compare the means of the different time points. Dunnett’s multiple comparison test was used for multiple comparison.

After one month following EVAR implantations, CTA was carried out to evidence a possible endoleak occurrence, and, on the basis of this evaluation, patients were stratified in two different cohorts: “Endoleak” and “No Endoleak”. The concentrations of all analyzed EV subpopulations were then compared between the two cohorts of patients. Total, as well as leukocyte-, platelet-, and endothelial-derived EVs were analyzed in patients developing (Endoleak) and not developing (No Endoleak) this EVAR complication (Figure 4).

**Figure 5:**
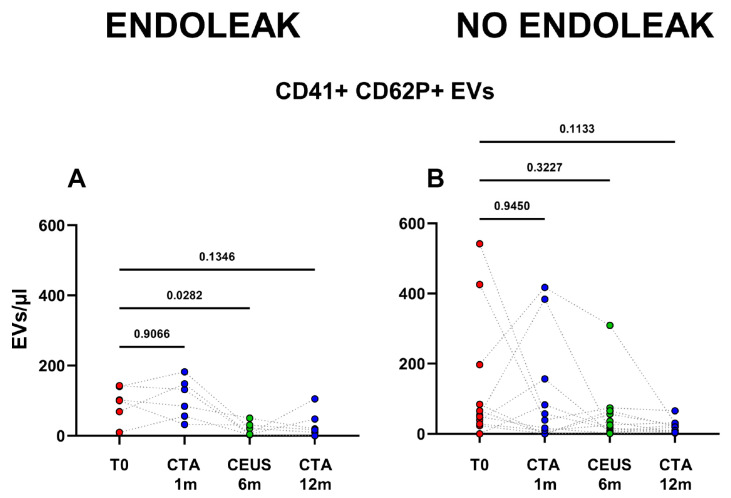
Patients were analyzed at baseline (T0), and after 1, 6, and 12 months following EVAR intervention, as established in the clinical protocol. In (**A**,**B**) dots represent numbers of peripheral-blood EVs stemming from activated platelets (CD41+ CD62P+) in the two cohorts of patients (Endoleak and No Endoleak). Each line connects the time points of a single donor. Repeated measures one-way ANOVA or a mixed-effects model was used to compare the means of the different time points. Dunnett’s multiple comparison test was used for multiple comparisons.

Interestingly, patients developing an endoleak showed a significant decrease in concentration of EVs stemming from activated platelets (CD41+ CD62P+) between the baseline condition (before EVAR) and after 6 months following EVAR intervention (*p* = 0.0282, Figure 5). Furthermore, in the same cohort a significant variation in total, as well as endothelial- (CD31+), activated-endothelial- (CD31+ CD62P+), and platelet- (CD41+) derived EVs were also detected when comparing values obtained after 1 month and after 12 months following EVAR implantation (Figure 4). In the cohort of patients that did not develop endoleaks, no significant variation in total, nor in endothelial- (CD31+), activated-endothelial- (CD31+ CD62P+), platelet- (CD41+), and activated-platelet (CD41+ CD62P+) derived EVs was detected, during the entire follow-up after EVAR implantation (Figure 4).

### 2.3. Endoleak Onset Induces the Release of EVs

**Figure 6:**
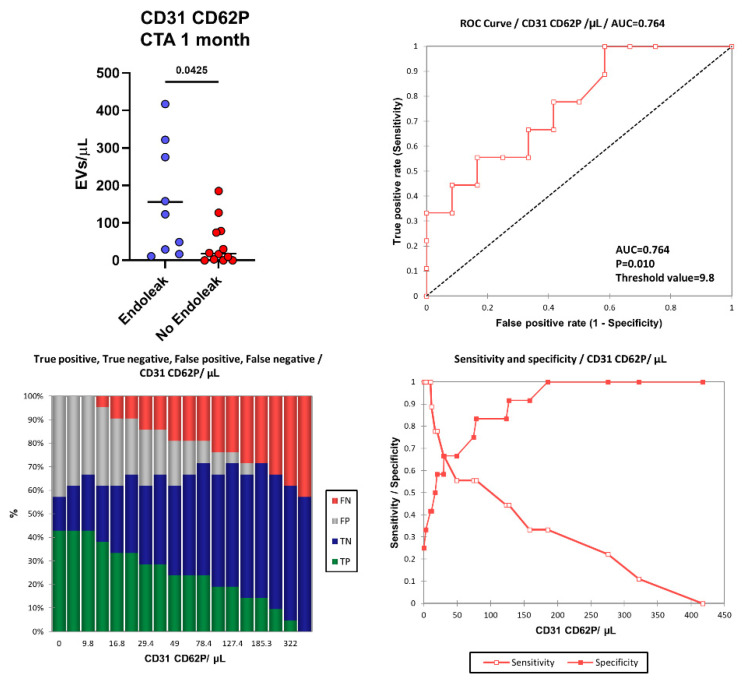
Number of CD31+ CD62P+ EVs were compared between the Endoleak and No Endoleak cohorts of patients after 1 month following EVAR intervention. Horizontal lines represent mean values. Statistical significance was calculated using non-parametric Mann–Whitney U-tests (two-tailed). Receiver operating characteristic (ROC) curve and performance plots, showing the reliability of CD31+ CD62P+ EVs used as early biomarkers to identify patients undergoing post-EVAR complications (i.e., endoleaks). TP = True Positive (green bar); TN = True Negative (blue bar); FP = False Positive (grey bar); FN = False negative (red bar). The threshold value of 9.8 CD31+ CD62P+ EVs/μL allowed us to obtain a sensitivity of 1.000, a specificity of 0.417, a PPV of 0.563, and an NPV of 1.000.

More interestingly, one month after EVAR intervention, in the “Endoleak” cohort of patients higher concentrations of EVs derived from activated endothelial cells (CD31+ CD62P+, *p* = 0.0425) were detected (155.87 vs. 45.36 EVs/µL) compared to the cohort of patients who did not develop endoleaks (Figure 6). Figure 6 shows the area under the ROC curve (AUC) for CD31+ CD62P+ EVs. Of note, patients presenting higher levels of CD31+ CD62P+ EVs (above 9.8 EVs/μL) one month after EVAR implantation were characterized by a considerably increased risk of developing endoleaks compared to patients with lower CD31+ CD62P+ EV concentrations. The model produced a significant power of discrimination (AUC = 0.764, *p* = 0.010, Sensitivity: 1.000, Specificity: 0.417, PPV: 0.563, NPV: 1.000) for patients developing endoleaks compared to subjects not developing endoleaks [63]. This indicates that the model is reliably able to distinguish between the “Endoleak” and the “No Endoleak” cohorts of patients. When clinical parameters (i.e., BMI) and EV levels were compared, no correlations were detected (data not shown).

## 3. Discussion

**Figure 7:**
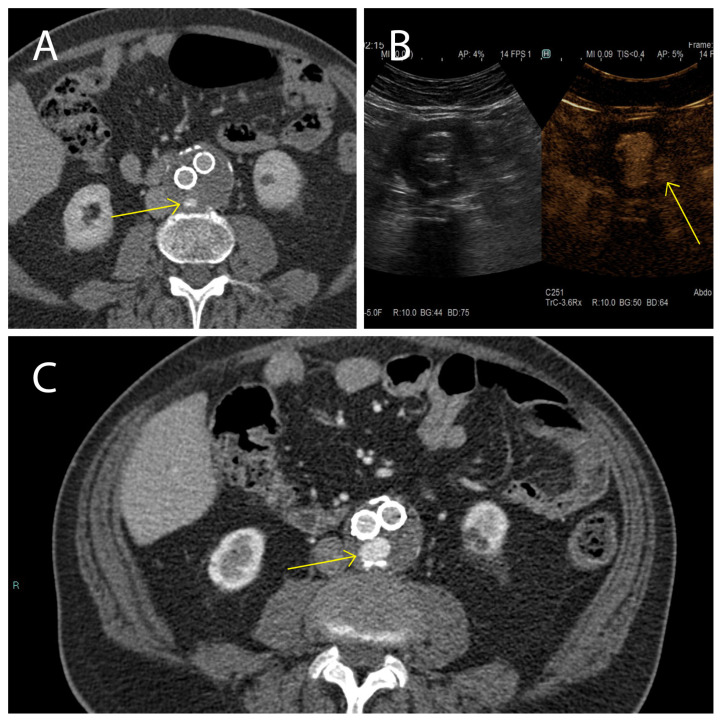
Presence and persistence of an endoleak. In these images a type II (yellow arrow) was evidenced using CTA 1 month after EVAR intervention, (**A**) monitored during the follow-up after 6 months using CEUS (**B**), and again using CTA 12 months after EVAR implantation (**C**).

Here we studied the fluctuations of EVs during the follow-up of patients affected by AAA and treated with EVAR, with particular attention to endoleak development, which is one of the major complications of this treatment. Our clinical data evidenced, that while in the cohort of patients not displaying endoleaks AAA size significantly decreased between 1 month and 12 months after EVAR intervention, in patients with the endoleak no AAA size variations were registered during the same period. These data confirm that in patients not developing endoleaks, a correct aneurysm repair process occurred. In the cohort of patients who developed the endoleak during the follow-up, a stable aneurysm diameter was associated with persistence of the endoleak itself [64]. Endoleak diagnoses are based on examination with CEUS and CTA during the post-EVAR follow-up (Figure 7). It must be underlined that these radiological analyses, except for the CEUS, expose patients to high doses of radiation, and are therefore not a particularly safe diagnostic strategy [4]. This point carries great weight if we consider that, as established by the latest ESVS guidelines [4], the follow-up period for these patients covers a time range of 5 years starting with the EVAR implantation. In such a context, it is relevant to identify new diagnostic/prognostic markers for the detection of endoleaks early after onset, which would be a safer diagnostic procedure than repeated CTA scans. As a matter of fact, the current ESVS guidelines underline that new strategies to stratify patients are needed to eventually reduce unnecessary EVAR imaging examinations during the follow-up [4]. In such a context, it has been widely demonstrated that EVs are clinically relevant diagnostic and prognostic biomarkers in cardiovascular diseases, as well as in many other clinical conditions, also providing insights into the underlying mechanisms [44,65,66,67,68,69].

It has been demonstrated that EVs derived from platelets and endothelial cells have pro-thrombotic and atherogenic potential and EVs stemming from the endothelium are associated with different cardiovascular diseases and have great potential as biomarkers in this context [44,70,71]. Furthermore, it is known that platelets are activated in AAA [72], and EVs stemming from activated platelets are involved in the pathogenesis of AAA [73]. It is known, that in patients affected by aortic aneurysm, platelet-derived EVs carry increased levels of ficolin-3, compared to healthy subjects, and these EVs were associated with aneurysm progression [74]. Ficolin-3 induces the activation of the complement system through the lectin pathway [75]. The complement system, in turn, mobilizes innate immunity, and thereby induces inflammation, which is the underlying cause of different cardiovascular disorders, including AAA [73,75]. In line with these findings, we observed a significant decrease in circulating EVs derived from platelets during the first 12 months after EVAR treatment for AAA, specifically between the first follow-up and 6 months after EVAR intervention. Such a decrease in EVs is therefore associated with a not correctly excluded AAA using EVAR. This condition is characterized by continuous thrombus remodeling and intense crosstalk between the coagulation cascade and the complement system [74]. Therefore, the site of the endoleak resembles a microenvironment characterized by constant bleeding [76,77,78]. Against that background, we hypothesize that EVs stemming from activated platelets undergo a prolonged “consumption-by-use” for sealing of the aneurysm sac. In other words, when the endoleak develops, activated platelets are recruited to seal the aneurysm sac resulting in an initial positive peak of EVs in the blood. When the platelets seal the sac and EVs reach the aneurysm sac from the blood, a progressive decrease in circulating EVs derived from activated platelets occurs. On the other hand, in the cohort of patients who did not develop endoleaks during the post-EVAR follow-up, no significant variations of EV counts were registered, demonstrating that in those cases, the aneurysm was correctly excluded using EVAR intervention. Overall, these data indicate that EVs actively participate in the pathophysiological events related to endoleak development in AAA patients treated with EVAR intervention.

More interestingly, we also observed that, one month after EVAR implantation, patients developing endoleaks showed higher concentrations of EVs derived from activated endothelial cells than patients who did not develop endoleaks. We also calculated a cut-off value for circulating EVs derived from activated endothelial cells. We were able to distinguish patients developing endoleaks early, i.e., one month after EVAR intervention, and with a high sensitivity. On the one hand these data suggest that the peripheral concentration of EVs stemming from the activated endothelium may have strong potential as biomarkers for the early diagnosis of endoleaks in EVAR treated AAA patients. On the other hand, this EV population may contribute to the pathophysiological crosstalk characterizing the sealing of the aneurysm sac. It has been demonstrated, in fact, that in thoracic aortic aneurysm and dissection, the related mechanical stretch induces release of EVs from smooth muscle cells, leading to an endoplasmic reticulum-stress resulting in endothelial dysfunction [79]. It is also known, that endothelial-derived EVs are associated with adiposity and subclinical CVD risk in a pediatric population [80]. The miRNA cargo of peripheral EVs changes in smoking young, healthy adults and these alterations contribute to smoking-related cardiovascular diseases [81]. Interestingly, it has been demonstrated that the progression of AAA is associated with the presence of antiphospholipid antibodies (aPLs), frequently related to autoimmune and cardiovascular diseases [82]. Those antibodies have also been associated with elevated concentrations of endothelial-derived EVs, increased levels of inflammatory markers, and AAA progression [82,83,84,85]. Altogether, these findings suggest a general mechanism related to the occurrence of inflammation in AAA onset and progression, involving EVs from activated platelet and endothelial cells.

## 4. Materials and Methods

### 4.1. Study Population and Design

We designed a monocentric, prospective, experimental, and non-pharmacological study focused on infrarenal AAAs.

A total of 25 patients were initially enrolled; 22 of them were finally included in this study, given that 3 patients with a previous cancer diagnosis were excluded from the analyses. These 22 patients (21 males and 1 female), aged between 65 and 87 years old, with an AAA diagnosis, and requiring EVAR intervention [4,5] were recruited by the Unit of Radiology of the “SS. Annunziata” Hospital (Chieti, Italy) between October 2019 and December 2020. For each enrolled subject completed an anamnestic questionnaire regarding general health conditions, cardiovascular risk factors, and medical therapies was completed. In all patients, eGFR-values ranged between 13.00 and 98.00 mL/min (Table S1).

A signed written informed consent, a minimum age of 60 years, and eligibility for EVAR intervention as a therapeutic approach were identified as inclusion criteria. Patients with known allergies to iodinate and/or ultrasonographic contrast medium, subjects affected by acute or chronic infectious conditions or a cancer diagnosis in the past 5 years, patients with untreated severe coronary artery disease (CAD), subjects not eligible for EVAR intervention, and subjects already treated for AAA with OSR/EVAR were excluded from the study. Patients dropped out from the study by voluntary withdrawal, when demonstrating poor compliance, or when they did not appear for the follow-up.

From each enrolled patient, peripheral blood was collected, using sodium citrate as an anticoagulant, for further EV analysis at four different time points:

- At the AAA diagnosis, eligible for EVAR;
- After 1 month folllowing EVAR implantation (at the same time point patients underwent a follow-up CTA of the abdominal aorta);
- After 6 months following EVAR implantation (at the same time point patients underwent a follow-up CEUS of the abdominal aorta);
- After 12 months following EVAR implantation (at the same time point patients underwent a follow-up CTA of the abdominal aorta).

In order to prevent possible variations in EV count after contrast-medium injection, and to analyze the EV baseline conditions, blood samples were collected before CTA or CEUS were performed.

After 1 month following EVAR implantation, patients were stratified into 2 groups: (1) patients with no endoleak evidence (*n* = 13), and (2) patients with evidence of an endoleak (*n* = 9). In all “Endoleak” patients a type II endoleak was diagnosed using CTA. As 3 patients originally sorted into the “Endoleak” cohort dropped into the “No Endoleak” cohort during the follow-up, and one patient belonging to the “No Endoleak” cohort withdrew from the study after the first follow-up, those patients were excluded from the time-point analyses.

Among the enrolled patients, no severe limb stenosis was diagnosed during the whole follow-up.

### 4.2. CT Scan Protocol

Abdominal aorta CTA was performed on a 128-slice multi-detector CT scanner (Somatom Definition AS+, Siemens Healthineers^®^, Erlangen, Germany), in a supine position, during inspiratory breath-holding. The field of view (FOV) was extended from the diaphragm to the femoral heads. The electronic window values were amplitudes (W) in a range of 1200–1600 Hounsfield Unit (HU) and window center levels (L) between 250 and 300 HU. The main scan parameters were tube voltage = 120 kVp, automatic tube current modulation (100–120 mAs), pitch = 0.8–1 mm, and matrix = 512 × 512. The scan protocol included a CT acquisition without contrast medium injection, followed by an angiographic acquisition with the following reconstruction parameters: slice thickness 1 mm, kernel B36F, and HeartView medium-ASA-window CT angiography to image the aorta; slice thickness 3 mm, kernel B30, and medium-smooth-window CT angiography to image the abdomen. During follow-up CTA, a venous acquisition 70 s after contrast medium injection was added. The bolus-tracking technique was used, positioning a region of interest (ROI) in the aorta, at the celiac trunk level, with a preferred HU threshold of 120 HU.

For the infusion protocol, a high concentration contrast medium (Iomeron 400 mg/mL—Iomeprol, Bracco Imaging^®^, Milano, Italy) was injected into the right median antecubital vein at a flow rate of 4 mL/s. With regard to having an iodine delivery rate (IDR) of 1.6 gI/s for better vascular imaging, the volume of contrast medium administered ranged from 60 to 120 mL depending on the flow and total infusion time.

### 4.3. CEUS Protocol

A follow-up CEUS was performed 6 months after EVAR implantation, using an ARIETTA 850 (Hitachi Healthcare^®^, Tokyo, Japan) ultrasound platform equipped with a Hitachi C251 convex probe (operating frequency 1–5 MHz). An ultrasound imaging contrast agent (Sonovue-Sulphur Hexafluoride, Bracco Imaging^®^, Milano, Italy) was used to enhance the abdominal aorta. We used a bolus of 2.4 mL (followed by a 5 mL bolus of saline solution) injected into the left antecubital vein with dynamic real-time acquisitions until 3 min after bolus injection.

### 4.4. Flow Cytometry Analysis of EVs

For each enrolled patient, two sodium citrate tubes (Becton Dickinson Biosciences-BD, San Jose, CA, USA, Ref 454387) were used to collect peripheral blood (PB) from a peripheral vein, through an 18G needle. The first tube was discarded to remove any vesicles produced as a result of the vascular damage induced by venipuncture. Flow cytometry EV staining and analysis was performed as previously described [66,86,87,88]. Briefly, the staining was carried out by adding the mix of reagents detailed in Table 2 to 195 µL of PBS; then 5 µL of whole blood were added to the mix. After 45 min of staining (RT, in the dark), 500 µL of PBS 1X were added to each tube, and 1 × 10^6^ events/sample were recorded using flow cytometry (FACSVerse, BD Biosciences, San Jose, CA, USA). The trigger threshold was placed on the channel in which the LCD emits (i.e. APC). MegaMix-Plus beads (Byocitex, Marseille, France) were used to roughly verify the placement of the gating on scatter parameters that was established in the beginning of the study on the scattered dot-plots [89]. Platelets were used as internal reference population as well. Data were analyzed using FACSuite v 1.0.6.5230 (BD Biosciences) software. EV concentrations were obtained using the volumetric count function, using the BD FACSVerse™ flow cytometer. The whole population of circulating EVs and the EV subtypes were identified as shown in Figure S1.

### 4.5. Statistical Analysis

Statistical analyses were performed using GraphPad Prism ver.9.0 (GraphPad Software Inc., La Jolla, CA, USA) and XLSTAT 2022 (Addinsoft, New York, NY, USA).

Differences in EV counts between two different groups were analyzed using Student’s t-test or the Mann–Whitney U-test, as appropriate. Data were expressed as mean ± standard deviation (SD) or as median. Repeated measures one-way ANOVA, or a mixed-effects model if missing values were present, were used to compare the different time points. Dunnett’s multiple comparison test was used for multiple comparisons. The ROC curve was calculated to assess the predictive role of circulating EV concentrations. A *p*-value of <0.05 was considered statistically significant.

## 5. Conclusions

Notably, quantitative and qualitative differences in terms of EV populations were demonstrated in patients developing endoleaks compared to non-complicated EVAR-treated patients. Our data also suggest that EVs derived from activated endothelial cells have great potential as a noninvasive and specific biomarkers from peripheral blood for the early identification of aortic stent-graft long-term complications (i.e., endoleaks).

If this evidence can be confirmed by larger studies in order to validate their clinical applicability, they could have relevant implications in clinical practice, possibly changing the diagnostic and prognostic approach in AAA patients. The identification of a precise cut-off able to diagnose the presence or absence of endoleaks with a unique peripheral blood sample in AAA patients treated with EVAR, could be a revolutionary approach in the follow-up of these patients, resulting in savings for the whole healthcare system and less radiation exposure for patients.

Circulating EVs, with their rising strong potential as liquid biopsy, could represent a valid biomarker able of supporting the radiological findings and data. The combinatorial information given by EVs and radiological imaging could improve diagnostic accuracy, leading to an earlier diagnosis of diseases. A conjugated approach of these two diagnostic fields brings our scientific attention to a new medical and translational research branch, whose aim is not only to diagnose but also to prevent disease: Radiovesicolomics [69]. This neologism describes a research field which uses radiological and flow cytometry data sources to create and collect models for data integration and prediction to evaluate the complex mechanisms of various pathologies. In this scenario, radiovesicolomics could bring the struggle against EVAR complications in patients treated for AAA to the next level: earlier, more specific, and more sensitive diagnosis in future could lead not only to a rapid therapeutic approach, but also to prevention before complications, such as endoleaks, manifest.

## Figures and Tables

**Table 1:** Long-term graft-related complications after EVAR – Adapted from open access ref. [4]. 2018, Elsevier B.V. on behalf of European Society for Vascular Surgery.

Complications	Definition	Estimated Frequency During 5-Year Follow-Up
Type I endoleak	Peri-graft flow occurring from attachment sites	5%
A	Proximal end of stent graft	
B	Distal end of stent graft	
C	Iliac occluder	
Type II endoleak	Peri-graft flow occurring from collateral branches to the aneurysm; inferior mesenteric artery (IIA) and lumbar arteries (IIB)	20–40%, 10% persistent at 2 years
	Categorised as early or late/delayed (before or after 12 months) and as transient or persistent (resolved or not resolved ≤ 6 months)	
Type III endoleak	Peri-graft flow occurring from stent graft defect or junction sites	1–3%
A	Leak from junctions or modular disconnection	
B	Fabric holes	
Type IV endoleak	Peri-graft flow occurring from stent graft fabric porosity < 30 days after placement	1%
Endotension	AAA sac enlargement without visualised endoleak	<1%
Migration	Movement of the stent graft in relation to proximal or distal landing zone	1%
Limb kinking and occlusion	Graft thrombosis or stenosis	4–8%
Infection	Stent graft infection	0.5–1%
Rupture	Aortic rupture	1–5%

**Table 2:** List of flow cytometry specificities and reagents.

Detection	Fluorochrome	Vendor	Ab Clone	Catalog	Amount per Test
Phalloidin	FITC	BD Biosciences	-	626267 (custom kit)	0.5 µL
CD41a	PE	BD Biosciences	HIP8	626266 (custom kit)	5 µL
CD31	PE-Cy7	BD Biosciences	WM59	626266 (custom kit)	5 µL
CD45	BV510	BD Biosciences	HI30	626266 (custom kit)	5 µL
LCD	APC	BD Biosciences		626267 (custom kit)	0.5 µL
CD62P	BV421	BD Biosciences	AK4	564038	3 µL

Keys: R-phycoerythrin (PE); PE-Cyanine 7 (Cy7), Brilliant Violet (BV). Becton Dickinson (BD) Biosciences (San Jose, CA, USA).

## Data Availability

The datasets generated during and/or analyzed during the current study are not publicly available due to the clinical and confidential nature of the material, but can be made available from the corresponding author on reasonable request.

## Supplementary Materials

The following supporting information can be downloaded at: https://www.mdpi.com/article/10.3390/ijms232416015/s1, Figure S1. Gating Strategy for analysis and subtyping of extracellular vesicles (EVs). (a) A platelet-free-area gate was drawn on a Forward Scatter-H/Side Scatter-H (FSC-H/SSC-H) dot-plot, using platelets as reference population. (b) The “Platelet-free-area” region was shown on a Phalloidin-H/Lipophilic Cationic Dye (LCD)-H dot-plot and EVs were identified as LCD positive/phalloidin negative events. (c) EVs (LCD+/Phalloidin-events) were analyzed on a CD45-H/CD31-H dot-plot and CD45+-events were identified as leukocyte-derived EVs. (d) A logical gate excluding all the CD45+-events was then obtained, and the resulting population (CD45−) was plotted on a CD31-H/CD41a-H dot-plot. CD31+ CD41a+-events were identified as platelet-derived EVs, while the CD31+ CD41a-compartment represented endothelial-derived EVs. Platelet-derived (e) and endothelial-derived (f) EVs were analyzed for their positivity to the activation marker CD62P. (g) The used gating is shown as a scheme. Table S1. Characteristics of patients included in the study at T0.
