# Supplementary material for: Circulating Extracellular Vesicles: Their Role in Patients with Abdominal Aortic Aneurysm (AAA) Undergoing EndoVascular Aortic Repair (EVAR)
Source: Int J Mol Sci. 2022 Dec 16;23(24):16015. doi: 10.3390/ijms232416015 (PMC9782915; doi:10.3390/ijms232416015)
Supplement: Supplementary file 1 [file ijms-23-16015-s001.zip › ijms-1996333-SI.pdf]

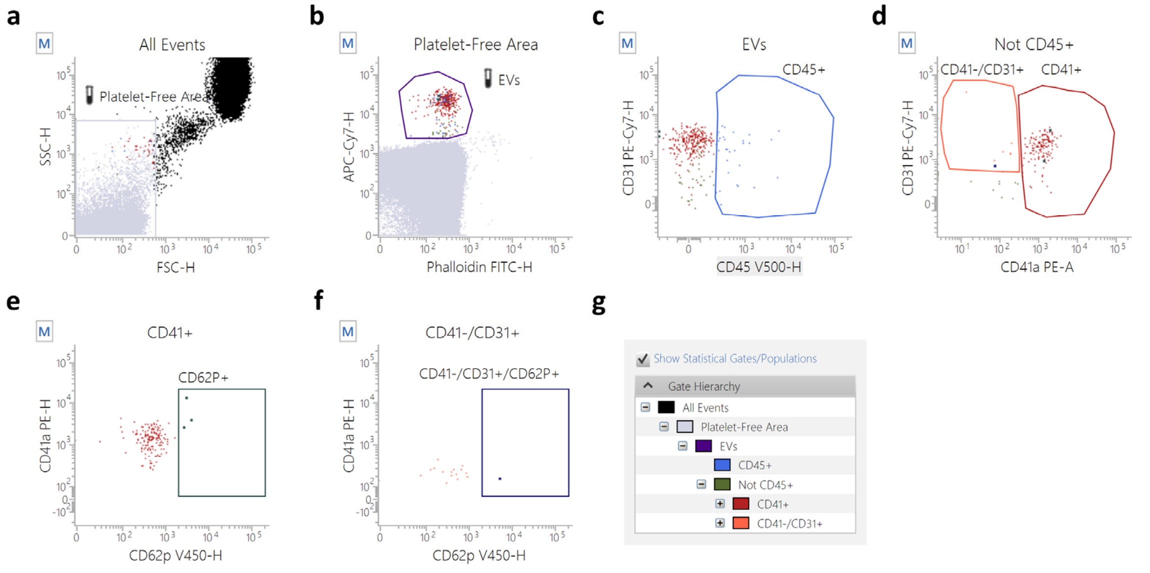

Figure S1. Gating Strategy for analysis and subtyping of extracellular vesicles (EVs). (**a**) A platelet-free-area gate was drawn on a Forward Scatter-H/Side Scatter-H (FSC-H/SSC-H) dot-plot, using platelets as reference population. (**b**) The "Platelet-free-area" region was shown on a Phalloidin-H/Lipophilic Cationic Dye (LCD)-H dot-plot and EVs were identified as LCD positive/phalloidin negative events. (**c**) EVs (LCD+/Phalloidin-events) were analyzed on a CD45-H/CD31-H dot-plot and CD45+-events were identified as leukocyte-derived EVs. (**d**) A logical gate excluding all the CD45+-events was then obtained, and the resulting population (CD45−) was plotted on a CD31-H/CD41a-H dot-plot. CD31+ CD41a+-events were identified as platelet-derived EVs, while the CD31+ CD41a-compartment represented endothelial-derived EVs. Platelet-derived (**e**) and endothelial-derived (**f**) EVs were analyzed for their positivity to the activation marker CD62P. (**g**) The used gating is shown as a scheme.

Table S1. Characteristics of patients included in the study at T0.

Patient No.	Weight (kg)	Height (cm)	BMI (kg/m²)	eGFR (ml/min)	Diabetes	High PA	Therapy	PLT (×10³/mm³)	PT (%)	Fibrinogen (mg/dL)
1	95	180	29.32	30.00	Yes	Yes	ASA + Clopidogrel	219	93.2	384
2	90	165	33.06	98.00	No	Yes	Clopidogrel	204	104.9	293
3	80	160	31.25	74.00	No	Yes	ASA	198	102.9	283
4	85	174	28.08	48.00	No	Yes	ASA	188	113.5	375
5	100	168	35.43	83.68	No	No	Warfarin + Clopidogrel + ASA	165	59.7	222
6	80	177	25.54	92.99	No	Yes	ASA	227	103.6	336
7	84	180	25.93	47.75	No	No	Dabigatran	194	84.4	359
8	70	167	25.10	55.00	No	Yes	ASA	193	92.7	271
9	83	170	28.72	75.00	No	No	-	236	81.7	230
10	70	169	24.51	54.00	No	Yes	-	212	95.4	366
11	78	170	26.99	65.00	No	No	ASA	201	91.9	307
12	86	167	30.84	75.70	Yes	Yes	ASA	204	99.8	315
13	100	163	37.64	59.32	No	Yes	ASA	159	87.4	366
14	77	156	31.64	58.00	No	Yes	ASA	188	98.6	285
15	66	164	24.54	81.00	No	Yes	ASA	185	93.7	349
16	75	170	25.95	68.27	No	Yes	ASA	335	74.3	291
17	64	152	27.70	88.23	No	Yes	ASA + Clopidogrel	167	97.9	282
18	74	166	26.85	51.00	No	Yes	-	156	103.4	251
19	75	170	25.95	82.00	No	No	ASA	142	112.8	236
20	100	170	34.60	36.00	No	Yes	Apixaban	230	89.4	407
21	91	172	30.76	13.00	No	Yes	ASA	389	83.7	570
22	90	170	31.14	55.00	Yes	Yes	ASA + Clopidogrel	259	107.7	330
